# X-ray photon correlation spectroscopy of protein dynamics at nearly diffraction-limited storage rings

**DOI:** 10.1107/S2052252519008273

**Published:** 2019-07-11

**Authors:** Johannes Möller, Michael Sprung, Anders Madsen, Christian Gutt

**Affiliations:** a European X-ray Free Electron Laser Facility, Holzkoppel 4, D-22869 Schenefeld Germany; b Deutsches Elektronen Synchrotron DESY, D-22607 Hamburg, Germany; c Department Physik, Universität Siegen, D-57072 Siegen, Germany

**Keywords:** materials science, structural biology, nanoscience, radiation damage, SAXS, storage rings, X-ray photon correlation spectroscopy, signal-to-noise ratio

## Abstract

This study explores the possibility of measuring the dynamics of proteins in solution using X-ray photon correlation spectroscopy (XPCS) at nearly diffraction-limited storage rings (DLSRs). We calculate the signal-to-noise ratio (SNR) of XPCS experiments from a concentrated lysozyme solution at the length scale of the hydrodynamic radius of the protein molecule. We take into account limitations given by the critical X-ray dose and find expressions for the SNR as a function of beam size, sample-to-detector distance and photon energy. Specifically, we show that the combined increase in coherent flux and coherence lengths at the DLSR PETRA IV yields an increase in SNR of more than one order of magnitude. The resulting SNR values indicate that XPCS experiments of biological macromolecules on nanometre length scales will become feasible with the advent of a new generation of synchrotron sources. Our findings provide valuable input for the design and construction of future XPCS beamlines at DLSRs.

## Abstract 2

How to optimize experimental setups to make BioXPCS measurements feasible at new generation synchrotron sources.

## 1. Introduction   

Dynamics in concentrated protein systems are of fundamental interest in fields such as protein crystallization (Durbin & Feher, 1996 ▸), phase separation (Anderson & Lekkerkerker, 2002 ▸), the glass transition (Cardinaux *et al.*, 2007 ▸) or diffusion in crowded environments (Ellis, 2001 ▸), to name just a few. These systems display relatively slow and heterogeneous dynamics ranging from microseconds to seconds on length scales from micrometres down to the single-particle nanometre scale. X-ray photon correlation spectroscopy (XPCS) is well suited to cover this length scale and time window, employing coherent X-ray beams and tracing fluctuations in X-ray speckle patterns (Sutton, 2002 ▸; Grübel *et al.*, 2008 ▸; Perakis *et al.*, 2017 ▸; Madsen *et al.*, 2018 ▸).

However, the highly intense X-ray beams of synchrotron storage rings can also cause considerable radiation damage to the samples. Atomic scale XPCS experiments use X-ray doses of MGy and beyond, which can lead to beam-induced dynamics even in hard-condensed-matter samples (Ruta *et al.*, 2017 ▸; Pintori *et al.*, 2019 ▸). Soft and biological matter samples are much more sensitive to radiation damage requiring flowing samples (Fluerasu *et al.*, 2008 ▸; Vodnala *et al.*, 2018 ▸) or scanning samples with optimized data-taking strategies (Verwohlt *et al.*, 2018 ▸). Typical critical X-ray doses for protein molecules in solution range from 7–10 kGy (BSA) to 0.3 kGy (RNase) after which a degradation of the small-angle X-ray scattering (SAXS) patterns becomes visible (Jeffries *et al.*, 2015 ▸). These doses are easily reached within milliseconds when using focused beams of modern synchrotron sources. While cryogenic cooling helps to prevent the diffusion of radicals in protein crystallography, such an approach is obviously impossible when studying the dynamics of proteins in solution.

The signal-to-noise ratio (SNR) in XPCS experiments ideally scales linearly with the coherent flux and thus with the source brilliance Br (Lumma *et al.*, 2000 ▸). The fastest accessible times scale with Br^2^, promising four orders of magnitude faster temporal resolution at the upgraded sources of ESRF and PETRA IV (Einfeld *et al.*, 2014 ▸; Weckert, 2015 ▸; Schroer *et al.*, 2018 ▸), which is one of the key drivers for XPCS at nearly diffraction-limited storage ring (DLSR) sources (Shpyrko, 2014 ▸). However, these arguments only hold if radiation damage can be mitigated. Thus, the question arises how much could XPCS experiments of biological/radiation-sensitive samples really benefit from the gain in brilliance of DLSRs? Here, we show that the combination of (i) larger coherence lengths, (ii) higher photon energy and (iii) the increased coherent photon flux yields an increase in SNR of up to one order of magnitude when compared with standard XPCS setups at today’s storage rings. We calculate, using the boundary conditions set by the maximum tolerable X-ray doses of a lysozyme solution, the XPCS speckle contrast, speckle intensities and the maximum number of images per illuminated spot. We conclude that future DLSRs hold the promise to measure the dynamics of biological samples at length scales of a single protein molecule.

## 2. XPCS on protein solutions   

XPCS experiments track fluctuations in X-ray speckle patterns yielding access to the intermediate scattering function *f*(*q*, τ) = *S*(*q*, τ)/*S*(*q*) by correlating intensities per detector pixel (Sutton, 2002 ▸). The measured signal in such experiments is the normalized intensity autocorrelation function (Sutton, 2002 ▸; Grübel *et al.*, 2008 ▸; Madsen *et al.*, 2018 ▸),
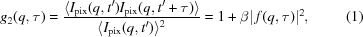
with β denoting the speckle contrast and 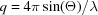 being the scattering vector, depending on the wavelength λ and the scattering angle 2Θ. The time delay between two consecutive time frames is denoted as τ, and 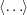 is the ensemble average over all equivalent delay times τ and pixels within a certain range of the absolute value |**q**|.

The scattering intensity per pixel from a protein solution is given by (Narayanan, 2008 ▸) 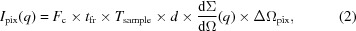
with *F*_c_ denoting the incident coherent flux (photons s^−1^), *t*_fr_ denoting the exposure time for one frame, *T*_sample_ denoting the sample’s transmission and ΔΩ_pix_ = (*P*/*L*)^2^ denoting the solid angle covered by a single pixel, with *P* being the pixel size and *L* being the sample-to-detector distance. In the following, we set the sample thickness *d*(*E*) to be equal to the absorption length of water *d*(*E*) = 1/μ(*E*) at each respective photon energy *E*, resulting in a transmission 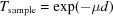 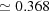. The differential scattering cross-section per unit volume or absolute scattering intensity in m^−1^ of a protein solution is defined as (Glatter & Kratky, 1982 ▸; Feigin & Svergun, 1987 ▸; Narayanan, 2008 ▸) 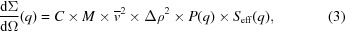
with *P*(*q*) the form factor, *S*_eff_(*q*) the effective structure factor and *C* the protein concentration. We will calculate the SNR for lysozyme as the model protein with a molar mass of *M* = 14.3 kDa and a specific volume of 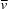 = 0.74 cm^2^ g^−1^. The scattering contrast Δρ follows from the chemical composition of lysozyme and shows almost no dependence on energy in the energy range of interest here. With these parameters, the absolute scattering intensity can be expressed as
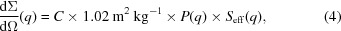
in good agreement with the measured values of 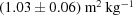 (Mylonas & Svergun, 2007 ▸).

**Figure 1:**
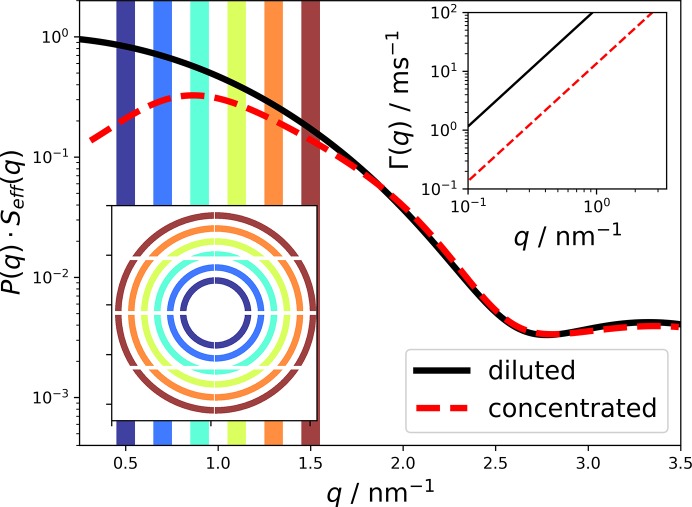
Modeled small-angle scattering intensity of a diluted [*P*(*q*), black line] and a concentrated lysozyme solution [*P*(*q*) × *S*_eff_, red dashed line]. The upper-right inset shows the expected relaxation rate Γ(q) as a function of *q* for both cases. The lower-left inset displays the field of view of an EIGER 4M detector, with the different regions of interest marked in color.

*P*(*q*) and *S*_eff_(*q*) are modeled following Möller *et al.* (2012 ▸), and displayed in Fig. 1 ▸ for a diluted (*C* = 10 mg ml^−1^) and a concentrated (*C* = 250 mg ml^−1^) lysozyme solution. The *q* values of interest are within *q* = 0.5–1.5 nm^−1^, which corresponds to length scales of 4–12 nm.

The dynamics of the low-concentrated protein solution can be described as Brownian diffusion with a single exponential autocorrelation function 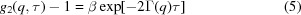
and relaxation rate 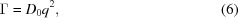
which is proportional to the Stokes–Einstein diffusion constant 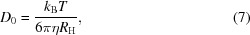
where *T*, η, *R*_H_ and *k*_B_ are the temperature, the viscosity of the suspending medium, the hydrodynamic radius of the protein and the Boltzmann constant, respectively. The *q*-dependent relaxation rate is plotted in the upper-right inset of Fig. 1 ▸ for diluted and concentrated lysozyme solutions. For the diluted case, we assume the viscosity of water and a hydrodynamic radius of *R*_H_ = 1.9 nm. We use an increased effective solution viscosity by a factor of 8.5 (Garting & Stradner, 2018 ▸) in order to illustrate the expected timescales for XPCS experiments on concentrated protein solutions. The time scales of interest range from 100 µs to s.

In practice, XPCS correlation functions are averaged over many pixels in a narrow range of *q* values. Typical regions of interest are sketched as colored areas in Fig. 1 ▸. The same set of regions is additionally depicted in the lower-left inset, showing the location of the corresponding pixels on an EIGER 4M detector for *E* = 8 keV and a sample-to-detector distance of *L* = 2 m. In the following, we will always calculate the SNR at the maximum of the structure-factor peak at *q* = 0.9 nm^−1^.

## 3. Signal-to-noise ratio   

The SNR for the autocorrelation function *g*_2_(*q*, τ) depends on the average intensity per pixel *I*_pix_, the contrast β, the number of pixels *N*_pix_, the number of frames *N*_fr_ and the number of repetitions *N*_rep_ via (Falus *et al.*, 2006 ▸) 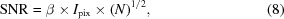
with *N* = *N*_pix_ × *N*_fr_ × *N*_rep_.

Considering *N*_fr_ = *T*/*t*_fr_, with *t*_fr_ being the single-frame exposure time and *T* being the total accumulated time for *N*_fr_ frames, yields in combination with equation (2), SNR ∝ *F*_c_(*t*_fr_ × *T*)^1/2^. This scaling implies that an increase in coherent flux *F*_c_ by one order of magnitude gives access to two orders of magnitude faster dynamics for the same SNR. However, this argument only holds when the available detectors are able to measure at the faster frame rates and the sample is capable of handling the increased photon flux. If a critical dose *D*_c_ exists, beyond which radiation-induced damage starts to degrade the sample, the longest overall exposure time *T* depends on *F*_c_ and the increase of coherent flux might be less, or not at all beneficial for studying radiation-sensitive samples.

The dose per second delivered to the sample depends on the photon flux as well as the photon energy, both of which also influence the achievable SNR. Here, we take all those parameters into account and calculate the benefit to the SNR from the increased coherent flux of DLSRs. We identify three parameters, which we will assume to be nearly free of choice over a wide range of values. These are the photon energy *E* = ℏ*c*/λ, the diameter *a* of the X-ray beam spot size on the sample, and the distance *L* between sample and detector. In the following, we will establish the dependencies of the different contributions on the SNR, and determine the optimal set of *a*, *E* and *L* values for an XPCS experiment using radiation-sensitive samples.

**Figure 2:**
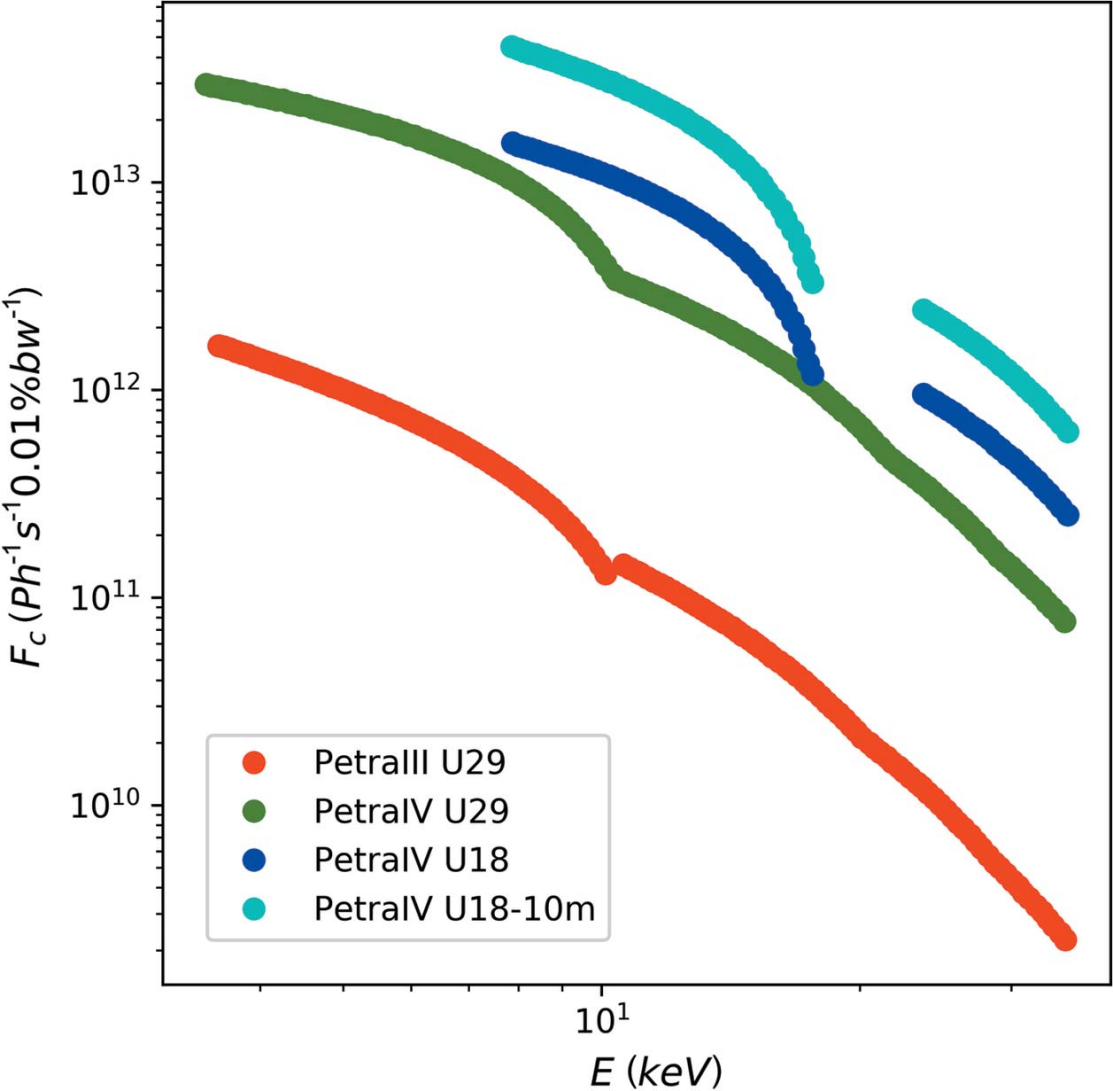
The coherent flux calculated from the brilliance taken from Schroer *et al.* (2018 ▸), assuming a bandwidth (bw) of 0.01%, corresponding to a Si(111) monochromator with Δλ/λ = 10^−4^.

Fig. 2 ▸ shows the expected increase of coherent flux as a function of photon energy for a U29 undulator (5 m length) at PETRA III and IV. Additionally, the case of a U18 with 5 and 10 m length will be investigated. The brilliance is taken from Schroer *et al.* (2018 ▸). From this, the coherent flux can be calculated as 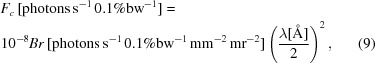
with bw denoting bandwidth. Using the referenced brilliance, we calculate the coherent flux for 8 keV at PETRA III as 3.8 × 10^11^ photons s^−1^. This is in good agreement with measured values of 2.3 × 10^11^ photons s^−1^, taking into account transmission effects of beamline components and optics. In the following, the actual coherent flux on the sample will be calculated by taking into account the same beamline transmission factor for all undulators.

### 3.1. Limitations due to radiation damage   

We assume that a critical dose *D*_c_, beyond which radiation-induced damage starts to degrade the sample, can be expressed as (Meisburger *et al.*, 2013 ▸) 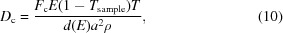
with *F*_c_ the photon flux on the sample, *d*(*E*) the energy-dependent sample thickness, *a* ^2^ the beam area, (1 − *T*_sample_) the sample absorption, *E* the photon energy and *T* the exposure time. From this, we derive the maximum number of frames which can be measured before radiation damage occurs to be 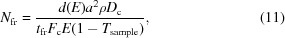
ignoring the latency time of the detector and absorption within the sample-container walls. The sample thickness *d*(*E*) is always adapted to the energy-dependent absorption length of water. One important conclusion from equation (11) is that the SNR scales via SNR ∝ *F*_c_(*N*_fr_)^1/2^ ∝ (*F*_c_)^1/2^ for radiation-sensitive samples. Moreover, with the scalings *d*(*E*) ∝ *E* ^3^ and *F*_c_ ∝ Br(*E*)/*E* ^2^, we also find the peculiar relation of *N*_fr_ ∝ *E* ^4^ favoring higher photon energies if a large number of frames is required.

We illustrate this with the example of a typical spot size for XPCS experiments of *a* = 4 µm, an exposure time of a single frame of *t*_fr_ = 1 ms and a critical dose limit for a concentrated lysozyme solution of *D*_c_ = 1 kGy.

**Figure 3:**
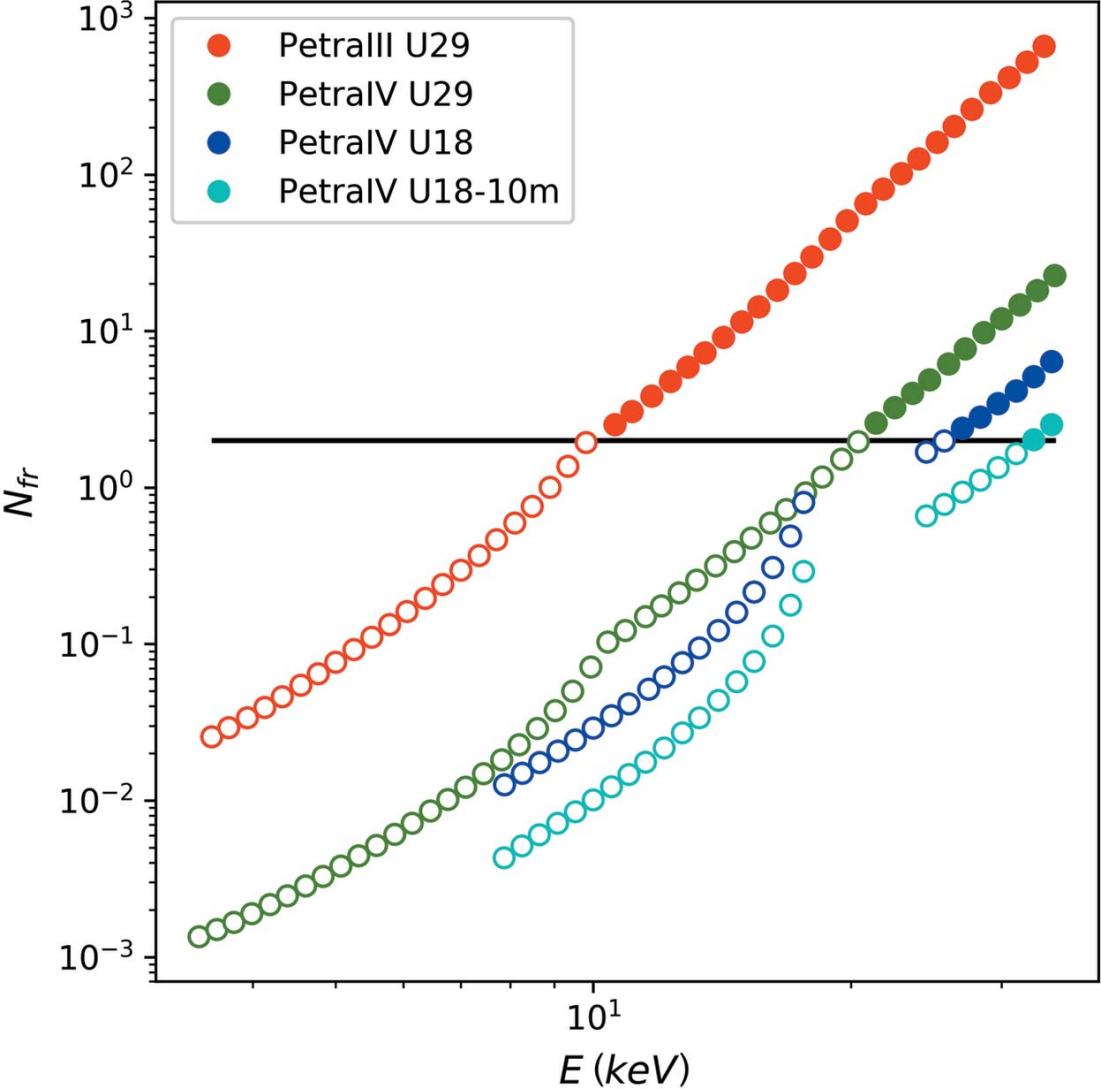
Maximum number of frames that can be measured on one spot before the onset of radiation damage for a lysozyme solution with beam size *a* = 4 µm and exposure time per frame of 1 ms. The horizontal black line depicts the threshold of at least two consecutive frames.

Fig. 3 ▸ displays the possible number of consecutive frames as a function of photon energy. A prerequisite for correlation spectroscopy is obviously that the number of consecutive frames is at least two (*i.e.* *N*_fr_ ≥ 2), indicated by filled symbols. The coherent flux of PETRA III already exceeds the critical dose after or during the first image, and beam damage is occurring between two images, for photon energies below 10 keV. Below this energy, an increase in coherent flux would therefore not be usable for XPCS experiments on protein samples. However, *N*_fr_ increases with photon energy because of the increasing absorption length of the X-rays. Effectively, the radiation dose is spread over a larger sample volume with increasing photon energy. However, many properties such as the speckle size, the coherent flux, and the longitudinal and transverse coherence lengths decrease with increasing photon energy. Therefore, the disadvantageous influence of these properties on the speckle contrast β and consequently on the SNR of XPCS experiments needs to be taken into account as well.

### 3.2. Speckle contrast β   

**Figure 4:**
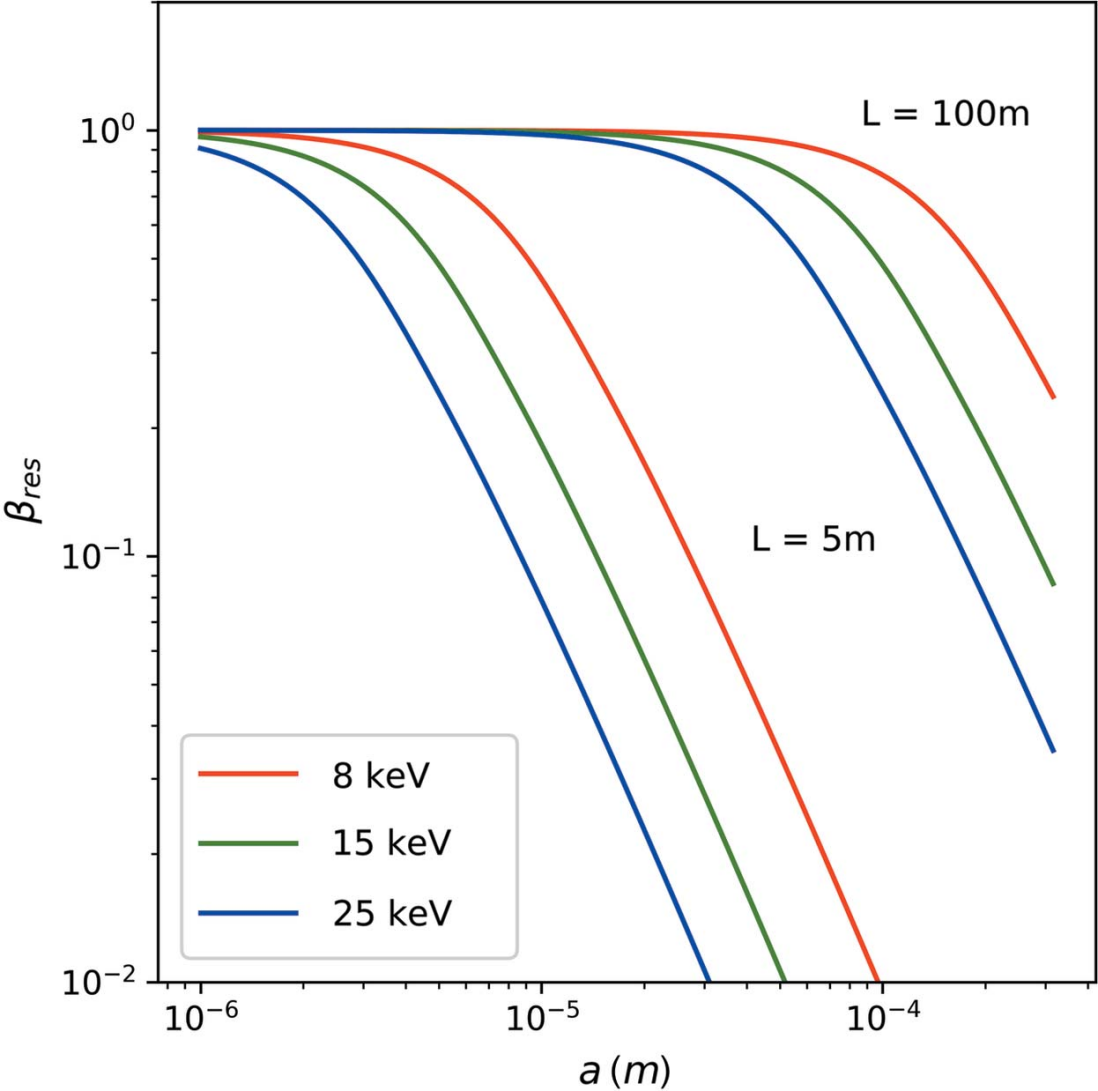
Speckle contrast β_res_ as a function of beam size *a* on the sample according to equation (13). Calculated for photon energies of 8 (red), 15 (green) and 24 keV (blue) and for sample-to-detector distances *L* = 100 m (top lines) and *L* = 5 m (bottom lines).

The speckle contrast depends on nearly all experimental parameters such as pixel size *P*, speckle size *S* ≃ λ*L*/*a*, beam size *a*, sample thickness *d*, wave vector transfer *q*, and the transverse and longitudinal coherence lengths. It can be written as a product,
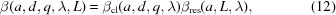
in which the first factor β_cl_ corresponds to the reduction of the contrast from unity caused by the finite coherence lengths in transverse and longitudinal direction. The second factor β_res_ corresponds to the finite angular resolution of the experimental setup. This results in a reduction of contrast if the pixel size of the detector *P* exceeds the size of the speckle *S*: 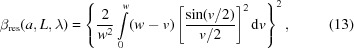
with *w* = 2π*Pa*/*L*λ = 2π*P*/*S*. Fig. 4 ▸ displays the speckle contrast β_res_ as a function of beam size *a* for sample-to-detector distances of *L* = 5 m and *L* = 100 m, pixel size *P* = 75 µm, and photon energies of 8, 15 and 25 keV. The maximum β_res_ is obtained in a high-resolution configuration with *S* ≥ *P* and scales as β ≃ λ^2^ *L* ^2^/*a* ^2^ *P* ^2^ in the low-resolution configuration, when 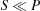. Therefore, XPCS experiments with large beam sizes require long sample-to-detector distances in order to resolve the smaller speckles.

**Figure 5:**
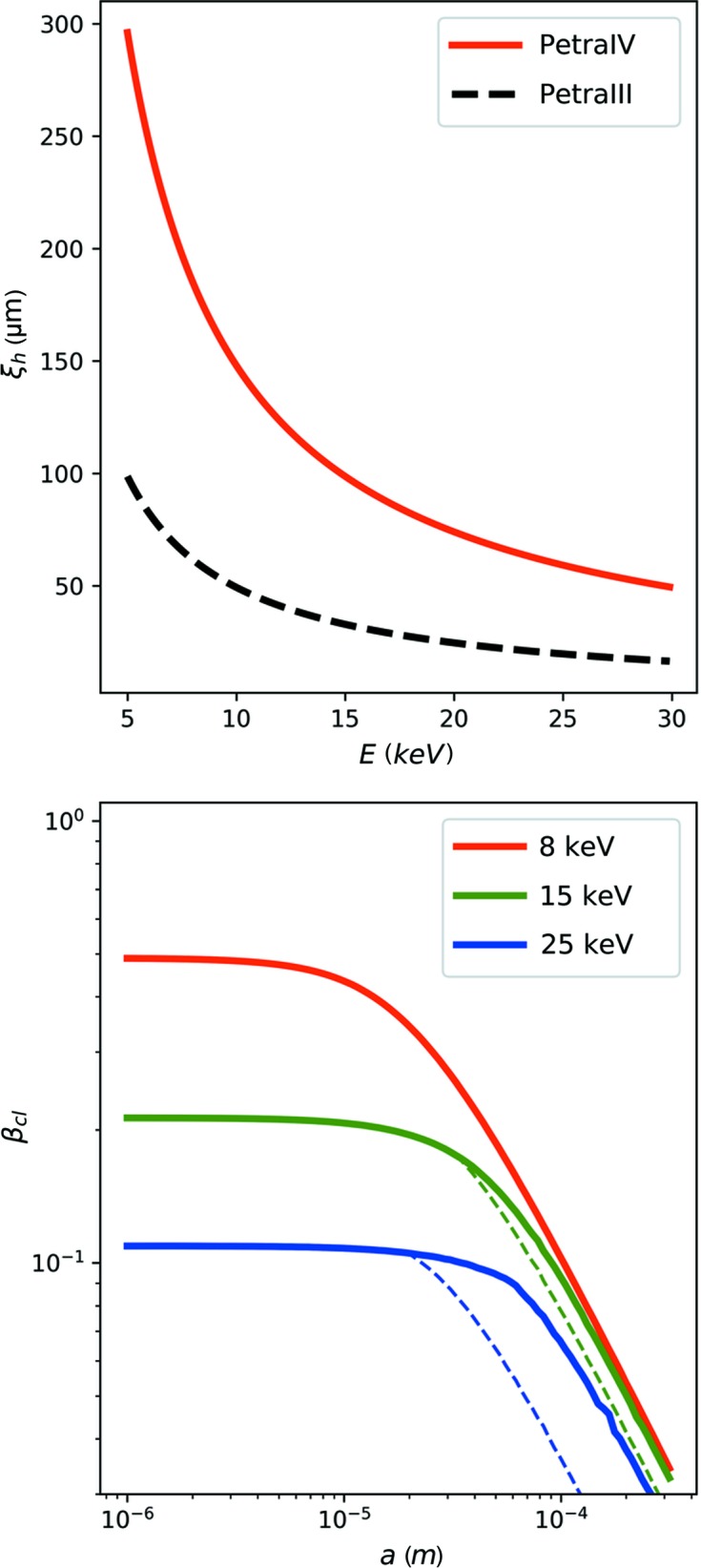
The horizontal coherence length calculated from the source properties of PETRA III and PETRA IV as a function of photon energy (top). The speckle contrast β_cl_ as a function of beam size calculated according to equation (14) [photon energies 8 keV (red), 15 keV (green) and 25 keV (blue)] (bottom). The dashed line corresponds to the horizontal coherence length at P10 PETRA III, the solid line represents the horizontal coherence length expected with PETRA IV. The *q* value is *q* = 0.9 nm^−1^ and the sample thicknesses are *d* = 1.0, 6.5 and 23 mm corresponding to the absorption length of water at the respective photon energies.

The dependence of β_cl_ on beam size *a*, sample thickness *d*, transverse coherence length ξ_h_, bandwidth 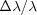 and *q* value is taken into account via (Sutton, 2002 ▸) 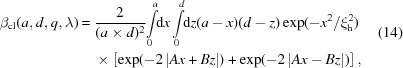
with 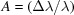 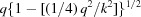, 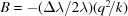 and *k* = 2π/λ. In the vertical direction, we assume a completely coherent beam. In the horizontal direction, a coherence length is estimated as 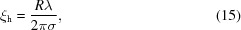
with *R* being the distance between the source and the beam-defining aperture, and σ being the RMS source size. With σ_h_ = 36 µm (P10, low-β source, 10 keV, *R* = 90 m), this results in a horizontal coherence length at *E* = 10 keV of ξ_h_ = 50 µm. A reduced horizontal source size at PETRA IV of σ_h_ = 12 µm would result in an increased horizontal coherence length of ξ_h_ = 148 µm at the same energy. These values reduce to 20 µm and 74 µm at an energy of *E* = 25 keV, respectively. The full energy dependence of ξ_h_ is shown in Fig. 5 ▸ (top).

Using a partially coherent source like a undulator for coherent-scattering experiments, a slice of the incident X-ray beam is required in order to obtain a nearly fully transversely coherent beam. Therefore, a beam-defining aperture is set to an opening size equal to the transverse coherence length. Smaller beam sizes can be achieved with additional focusing elements. For our calculations, we will consider the resulting focused beam as fully coherent with a ξ_h_ equal to the beam size. For larger beam sizes, ξ_h_ is calculated following equation (15).

The temporal or longitudinal coherence length can be calculated as 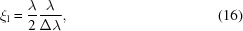
depending on the bandwidth of the monochromator used [Δλ/λ ≃ 1.4 × 10^−4^ for an Si(111) monochromator and Δλ/λ ≃ 3 × 10^−5^ for Si(311)].

The results for β_cl_ as a function of beam size and X-ray energy are shown in Fig. 5 ▸ (bottom) for a *q* value of *q* = 0.9 nm^−1^, corresponding to the peak of the structure factor shown in Fig. 1 ▸.

We observe a reduction in speckle contrast with increasing beam size and a reduced contrast for smaller beam sizes as a function of photon energy. Both reductions can be explained by the scattering volume, defined by spot size *a* and sample thickness *d*, exceeding the coherence volume defined by the longitudinal and transverse coherence lengths.

### 3.3. Number of pixels   

Changing the photon energy and sample-to-detector distance has direct implications on the number of pixels which can be covered within an area of a certain *q* range. The scattering signal may be in a circular region of interest on the detector of width Δ*q* and radius *q*. In the SAXS regime, *q* = (4π/λ)Θ and Δ*q* = (4π/λ)ΔΘ, and the diffraction ring has a width on the detector of Δθ × *L* and a circumference of 2π(2Θ)*L*. The number of illuminated pixels is thus
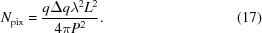
However, if the size of the speckles exceeds the size of the detector pixel *S* > *P*, the number of independent detecting pixels decreases, following in this case
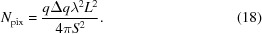



## 4. XPCS of protein solutions   

Having established the dependence of the SNR on the experimental parameters, we can use the expression 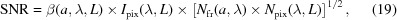
to characterize the influence of the improved brilliance of the new generation of X-ray sources on XPCS experiments with radiation-sensitive samples.

**Figure 6:**
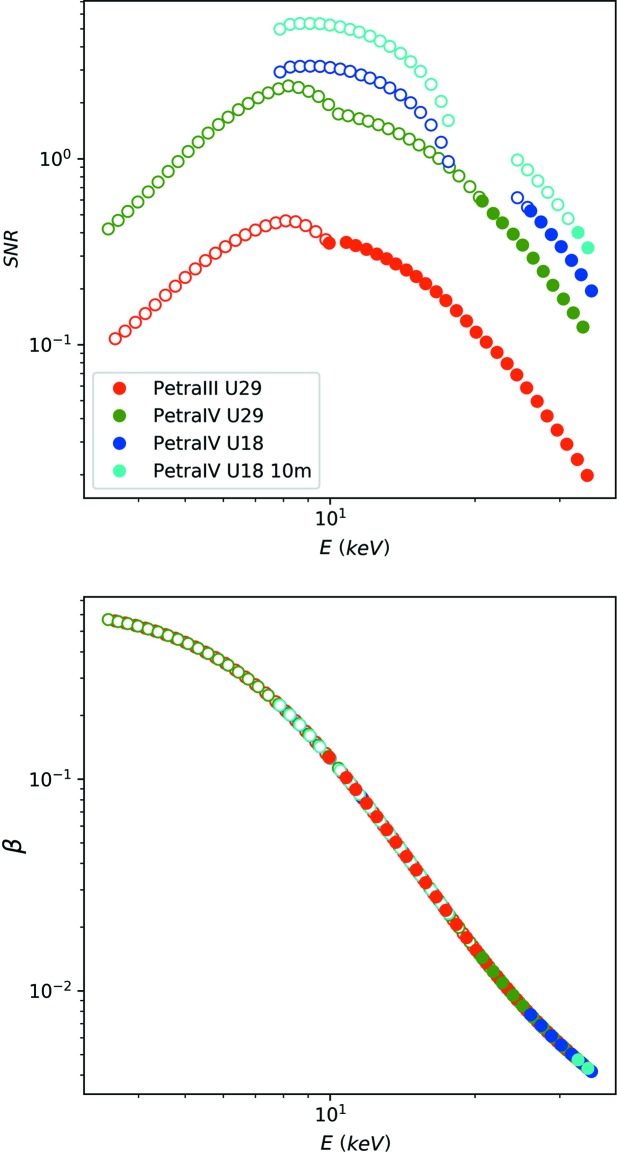
The SNR calculated as a function of photon energy for a setup of *a* = 4 µm, *L* = 2 m, for different undulators (top). The open symbols correspond to experimental conditions which are not accessible because of beam-damage effects. The speckle contrast of this setup as a function of photon energy (bottom).

In Fig. 6 ▸, we display the SNR for a standard XPCS setup. We assume that an EIGER 4M detector (Johnson *et al.*, 2014 ▸) is used, with a sample-to-detector distance of *L* = 2 m, which corresponds at a photon energy of *E* = 8 keV to the inset of Fig. 1 ▸. In order to match the speckle size to the pixel size, an X-ray spot size of *a* = 4 µm is required, corresponding to the calculations shown in Fig. 3 ▸. Further parameters are shown in Table 1 ▸.

The red data points correspond to the photon-beam properties of PETRA III, and the green, blue and cyan points correspond to the improved coherent flux *F*_c_ offered by PETRA IV with different undulators. As can be seen, the increasing coherent flux offers theoretically improved SNR values of more than one order of magnitude. However, as marked with open symbols, the highest theoretically possible SNR of each configuration corresponds to experimental conditions where the critical dose limit of the sample is reached within two sequential acquisitions (*i.e.* *N*_fr_ ≤ 2), and hence the sample would suffer from radiation-damage effects during the measurement. Therefore, the maximum increase in SNR can not be reached in practice for this setup, and the upgrade to PETRA IV would not lead to a significant increase in SNR.

Data points which correspond to beam conditions where at least two sequential acquisitions are possible are displayed as filled symbols. It is evident that higher beam energies with thicker samples would ease the effect of a higher flux and make XPCS experiments possible also at a standard configuration (*L* = 2 m, *a* = 4 µm). However, as displayed in Fig. 6 ▸ (bottom part), this also results in much reduced speckle contrasts and, therefore, the beneficial effect of an increased coherent flux on the SNR is largely lost.

### 4.1. Optimizing the experimental setup   

**Figure 7:**
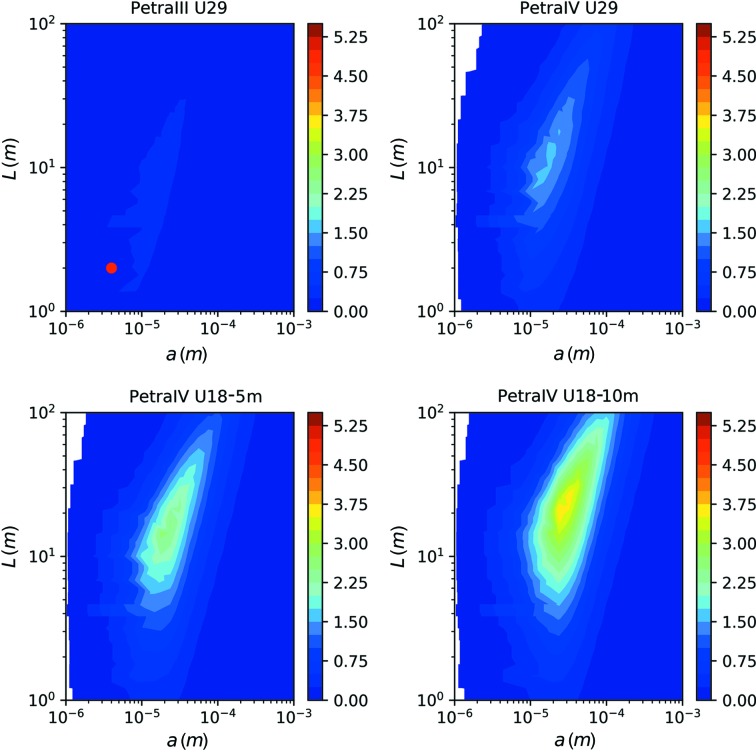
The maximum value of SNR as a function of sample-to-detector distance *L* and beam size *a* for DLSRs. The highest SNRs are SNR_P3_ = 0.4; SNR_P4_ = 1.7; SNR_P4U18-5m_ = 2.6; and SNR_P4U18-10m_ = 3.7.

In order to use the increased coherent flux for XPCS experiments, one has to adapt the experimental setup in terms of focusing, photon energy and sample-to-detector distance. Therefore, we repeat the previously presented calculations for a set of different beam sizes *a* and sample-to-detector distances *L*. At each point in the *a*–*L* plane, the SNR is calculated as a function of photon energy and the maximum is plotted. However, only values are considered which correspond to *N*_fr_ ≥ 2 at 1 ms exposure. The maximum SNR for each pair of *a* and *L* values is displayed in Fig. 7 ▸.

It can be seen that the previously discussed setup with a small beam and large speckle (marked by a red dot) does not give the best SNR, already evident in the case of PETRA III. With a sample-to-detector distance of *L* = 5.5 m and an X-ray spot size of *a* = 9 µm, the expected SNR increases by 25%.

However, in the case of PETRA IV (U18-10m), an overall increase in SNR by about one order of magnitude can be achieved, without exceeding the critical radiation dose of the sample. This setup would feature a sample-to-detector distance of *L* = 26 m and a spot size of *a* = 24 µm at *E* = 14.7 keV.

The resulting parameters for the optimized experimental setups are summarized in Table 2 ▸ for each of the considered undulators. We note that for higher coherent-flux setups, the optimized setups feature an increase in beam size *a*, sample-to-detector distance *L* and photon energy *E*.

As a general trend, it is evident that the sample volume, spanned by the sample thickness *d* and spot size *a*, needs to be increased when the coherent flux increases. In order to compensate for the consequently decreasing angular speckle size, the sample-to-detector distance needs to increase so that the speckle size can maintain its value of *S* ≃ 75 µm, matching the pixel size. However, it can be seen that one can still observe a decrease in speckle contrast β, even though the speckles have the same size on the detector for all four setups presented. This effect is due to the second contribution to the speckle contrast β_cl_, see equation (14), originating from the limited longitudinal coherence length of the X-ray beam.

### 4.2. Si(311) monochromator   

Here, we investigate how an additional increase of the longitudinal coherence length by using a Si(311) monochromator benefits the achievable SNR. We repeat the previous calculations, however, with a reduced bandwidth of 3 × 10^−5^ and a reduced flux compared with the Si(111) calculations by 74%.

**Figure 8:**
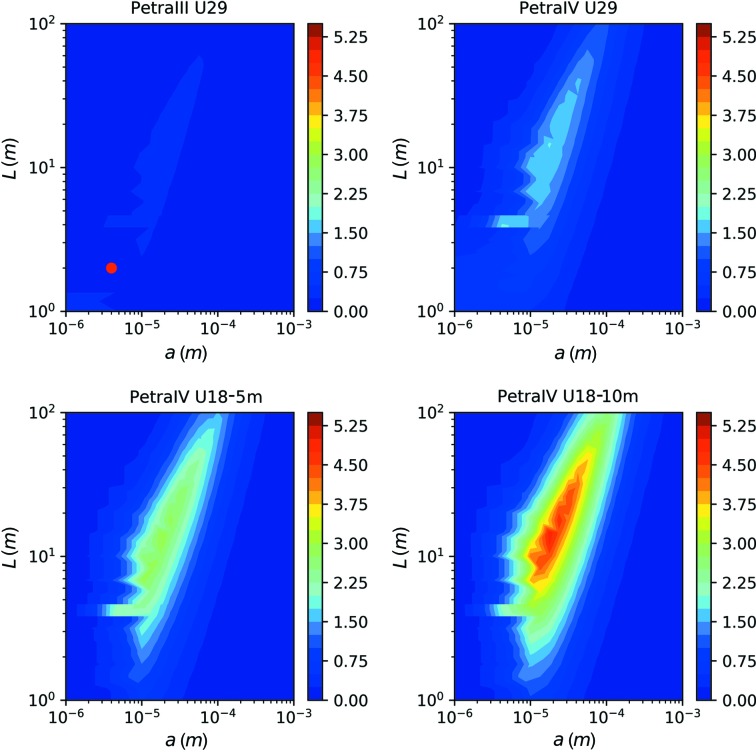
The best combination of sample-to-detector distance *L* and beam size *a* for DLSRs using an Si(311) monochromator.

The resulting SNRs are displayed in Fig. 8 ▸ and Table 3 ▸. We find that the use of a Si(311) monochromator improves the SNR by an additional 30% compared with the Si(111), thus leading to an overall SNR gain of a factor of 13 when comparing PETRA III with PETRA IV. Interestingly, the achievable SNR at PETRA III decreases when a Si(311) monochromator is used instead of a Si(111), showing that XPCS measurements at existing synchrotron sources can be considered as flux limited, whereas an increase in SNR can be noted for all three undulator types studied at PETRA IV.

### 4.3. Multiple-frame XPCS and two-time correlation functions   

It becomes evident that with SNR values ∝ 3–5, XPCS measurements from protein solutions are possible at DLSRs with adapted experimental setups. As a direct consequence of the presented results, the optimized data-acquisition scheme differs from conventional XPCS measurements. Instead of taking many hundreds to thousands of images at one spot, the scheme with maximum SNR for protein XPCS consists of ‘double-shot’ exposures. Therefore, a full-correlation function from one spot on the sample cannot be measured, but rather only one data point of *g*_2_ for each illuminated sample spot. Consequently, the correlation function would be constructed from many such double-shot exposures, each on a new sample spot and with a different delay time τ between the two frames (see *e.g.* Verwohlt *et al.*, 2018 ▸). The required sample volume therefore scales with the desired number of data points of *g*_2_.

**Figure 9:**
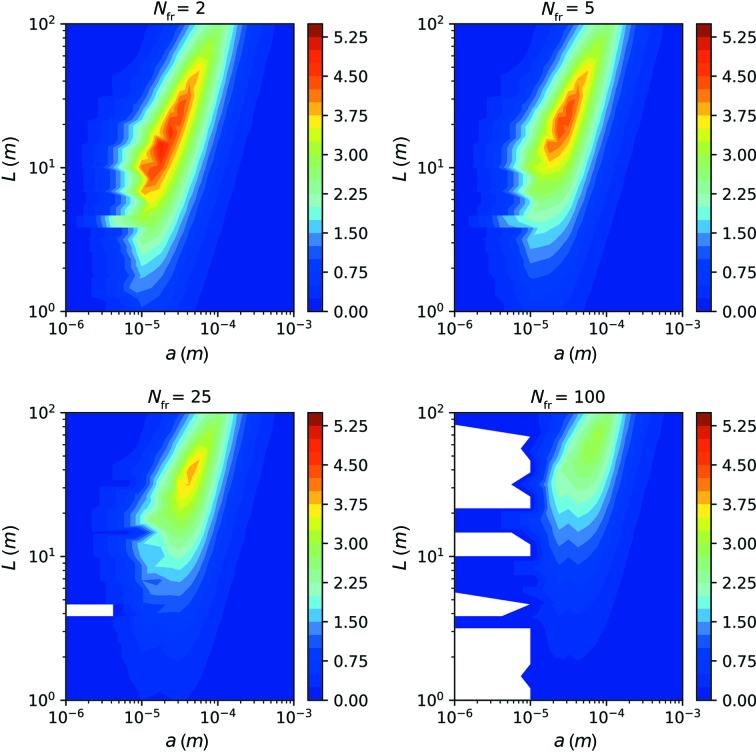
The maximum value of the SNR as a function of sample-to-detector distance *L* and beam size *a* for PETRA IV (U18-10 m), for *N*_fr_ = 2, 5, 25 and 100 frames taken on the same sample spot.

However, this acquisition scheme is not suitable for samples displaying heterogeneous dynamics or aging effects. In such cases, a movie-mode acquisition scheme with more than two frames per spot is needed. Fig. 9 ▸ displays the resulting SNR values in the *a*–*L* plane if the minimum number of frames is set to *N*_fr_ = 2, 5, 25 or 100 (for the case of PETRA IV U18-10m).

We find that with an increasing number of images, *N*_fr_, the value of the maximum SNR decreases only slightly, and its position in the *a*–*L* plane shifts towards larger beam sizes *a* and larger sample-to-detector distances *L*. For realizing the higher number of frames, an increase in the photon energy and in the beam size is required [from equation (11) we find the scaling *a* ∝ (*N*_fr_)^1/2^]. The resulting degradation of speckle contrast is partially counterbalanced by improving the angular resolution via a larger sample-to-detector distance. For example, for *N*_fr_ = 100 frames, the optimum SNR is still 3.2 at *a* = 75 µm, *L* = 82 m and *E* = 15.4 keV. Generally speaking, we find at the maximum of the SNR a scaling of *L* ∝ *a* ∝ [*N*_fr_Br(*E*)]^1/2^.

In practice, the realization of a beamline with up to 100 m sample-to-detector distance, which would also require a detector with a very large number of pixels, is challenging. However, it can be seen from Fig. 9 ▸ that at shorter sample-to-detector distances *L* the SNR is still significantly larger than 1 for *N*_fr_ ≤ 100. Therefore, we investigate SNR optimization if the length of the beamline is a fixed value of *L* and if a certain number of frames *N*_fr_ is required to track the physics of the protein solution.

**Figure 10:**
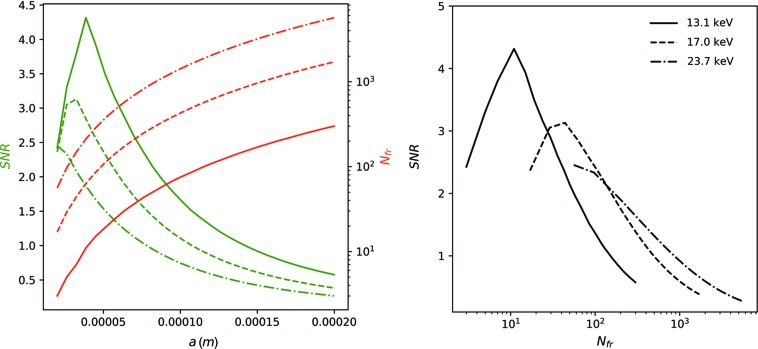
SNR (green lines) as a function of beam size *a* for photon energies of 13.1, 17.0 and 23.7 keV (solid, dashed, dashed–dotted lines, respectively) (top). Red lines indicate the maximum number of possible frames. SNR as a function of the maximum number of frames displayed for the same photon energies (bottom).

We demonstrate this by fixing the sample-to-detector distance to *L* = 30 m, which is already available at specialized ultra-small-angle scattering beamlines (see *e.g.* Möller *et al.*, 2016 ▸; Zinn *et al.*, 2018 ▸), and using a Si(311) monochromator. We plot both the SNR and the maximum number of possible frames as a function of beam size *a* (Fig. 10 ▸, top) for photon energies of *E* = 13.1 keV (solid), *E* = 17.0 keV (dashed) and *E* = 23.7 keV (dashed–dotted). Fig. 10 ▸ (bottom) displays the same data but plotted as SNR as a function of *N*_fr_ for the different photon energies. The benefit of using even higher photon energies than 13 keV for multi-frame acquisitions is obvious as it allows either an increase of the SNR value at fixed *N*_fr_ or the ability to record more images at a fixed value of the SNR. We find that with the source parameters of PETRA IV (U18-10m) the resulting values of the SNR can be well above 1, even for several hundreds of frames recorded. Specifically, we may take the example of *N*_fr_ = 100 and find an SNR value of 2.5 at 17 keV. The best combination of photon energy and spot size depends on the required number of frames, *i.e.* the timescales which are investigated. It can be seen that higher energies are beneficial in order to record multi-frame acquisitions below the radiation-damage threshold and with high SNR. Additionally, the higher energies shift the scattering angles to lower values, which consequently reduces the size of the detector required.

## 5. Conclusion   

We determined the signal-to-noise ratios for XPCS experiments of a concentrated lysozyme solution at length scales of the hydrodynamic radius of a single protein molecule. We showed that by adapting the experimental setup, XPCS measurements can be performed below the radiation-damage threshold and with strongly increased SNR. The results show that the SNR values can be increased up to one order of magnitude at future upgraded storage rings when compared with existing facilities.

With this, the required measuring time for multi-frame acquisitions would reduce by two orders of magnitude making dynamic studies of protein solutions at nanometre length scales feasible. However, in order to take full advantage of the properties of the future sources, XPCS experiments require experimental setups with larger beam sizes and longer sample-to-detector distances than usually available at standard XPCS beamlines. We showed that the optimal photon energy for soft-matter double-shot XPCS measurements at the upgraded storage ring PETRA IV will be between 12.5 and 14 keV, with sample-to-detector distances in the range of 8 to 15 m. Additionally, we showed that multi-frame acquisitions on protein solutions are possible with up to several hundreds of frames by further increasing the photon energy and increasing the length of the beamline (*E* ≥ 17 keV, *L* ≥ 30 m).

We hope that this study shows the opportunities offered by nearly diffraction-limited storage rings and may additionally serve as a guide for the design of soft-matter XPCS beamlines.

## Figures and Tables

**Table 1:** Parameters fixed for the calculations of the SNR

*q*	0.9 nm^−1^
Δ*q*	0.1 nm^−1^
*C*	250 mg ml^−1^
*P*(*q*) × *S*_eff_(*q*)	∼0.3
*D*_c_	1000 J kg^−1^ = 1 kGy
*P*	75 µm
*t*	1 ms

**Table 2:** Optimized setup for *N*_fr_ = 2 using a Si(111) monochromator

Parameters	U29 (PIII)	U29	U18-5m	U18-10m
SNR	0.4	1.7	2.6	3.7
Beam size *a* (µm)	7.5	13.3	17.7	23.7
Sample-to-detector distance *L* (m)	3.8	10.0	14.7	21.5
Beam energy *E* (keV)	8.1	12.2	13.6	14.7
Coherent flux *F*_c_(photons s^−1^)	2.1 × 10^11^	1.5 × 10^12^	3.4 × 10^12^	7.2 × 10^12^
Contrast β	0.20	0.12	0.10	0.08
Speckle size *S* (µm)	78	76	75	76
Intensity per pixel *I*_pix_ (photons ms^−1^)	2.3 × 10^−3^	8.0 × 10^−3^	1.2 × 10^−2^	1.5 × 10^−2^
No. of pixels in *q* range *N*_pix_ (*M*)	0.4	1.3	2.2	4.2
No. of frames *N*_fr_	2	2	2	2
Sample thickness *d* (mm)	1.0	3.6	4.9	6.3
Exposed sample volume (nL)	0.05	0.6	1.6	3.5

**Table 3:** Optimized setup using a Si(311) monochromator

Parameters	U29 (PIII)	U29	U18-5m	U18-10m
SNR	0.37	1.8	3.0	4.9
Beam size *a* (µm)	7.5	10.0	13.3	17.8
Sample-to-detector distance *L* (m)	5.6	8.3	10.0	14.7
Beam energy *E* (keV)	12.5	14.1	12.5	13.8
Coherent flux *F*_*c*_(photons s^−1^)	1.5 × 10^10^	2.9 × 10^11^	1.1 × 10^12^	2.3 × 10^12^
Contrast β	0.23	0.20	0.23	0.20
Speckle size *S* (µm)	74	73	74	74
Intensity per pixel *I*_pix_ (photons ms^−1^)	3.0 × 10^−3^	3.9 × 10^−3^	6.9 × 10^−3^	9.1 × 10^−3^
No. of pixels in *q* range *N*_pix_ (*M*)	0.06	0.7	1.3	2.2
No. of frames *N*_fr_	69	8	3	3
Sample thickness *d* (mm)	3.8	5.5	3.8	5.2
Exposed sample volume (nl)	0.21	0.55	0.7	1.6
